# Contact Dermatitis

**Published:** 1952-04

**Authors:** Clifford D. Evans

**Affiliations:** Consultant Dermatologist, United Bristol Hospitals


### CONTACT DERMATITIS

BY

CLIFFORD D. EVANS, O.B.E., M.B., B.Ch.

*Consultant Dermatologist, United Bristol Hospitals*

A contact or exogenous dermatitis is one in which an external irritant is the most important cause; but a constitutional factor undoubtedly also plays a part, and accounts for the fact that only a certain proportion of a community, in contact with the same irritant, develops dermatitis. Most cases of contact dermatitis clear up quickly when the irritant is removed, but in some the rash persists and spreads to other areas because the patient has a constitutional tendency to develop eczema. Contact dermatitis is responsible for a great loss of manpower in industry, and causes disability amongst the general population, especially in housewives.

The clinical appearances and histopathology of patches of eczema or dermatitis are identical; the former term is commonly used when constitutional factors are thought to be responsible for the condition, and the latter when external factors predominate. Unfortunately *dermatitis* suggests occupational dermatitis to the worker, and it is advisable to avoid the use of this name whenever possible, unless an occupational cause is likely, e.g. seborrhoeic eczema instead of seborrhoeic dermatitis. The first clinical finding is erythema of the affected area. Papules appear and then vesicles which burst and give rise to weeping. Infection may then supervene or the exudate may dry up and leave a scaling condition. Irritation is a prominent symptom and the rubbing and scratching cause thickening or lichenification of the skin.

The irritants can be divided into primary and sensitizing irritants. The primary ones are those which, used repeatedly, or in sufficient concentration, will affect the skins of most people. Examples of these are the strong acids, alkalis and soaps, degreasing agents such as petrol, lime and cement. Sensitizing irritants are those to which the skins of some people become sensitive after one or many contacts. Primulas, aniline dyes, turpentine, penicillin and streptomycin, antiseptics, and elastoplast are a few well-known substances of this group. Almost any chemical can sensitize the skin and cause dermatitis in a small proportion of people, but it is often very difficult to convince a patient that his rash has been produced by something which he has been handling for many years, and the patch test, if positive, is helpful in these cases. Primary irritants probably account for about eighty per cent of cases of contact dermatitis.

Certain factors are commonly found to precipitate an attack. The aged, whose skin is becoming dry and atrophic, are liable to develop contact dermatitis; increased exposure to the irritant occasioned by longer working hours may be the cause; constitutional disturbances, such as occur at puberty, the menopause, or with psychological trauma, may precipitate a breakdown. In some cases dermatitis develops on returning to work after a period away from it. The skin appears to have lost some immunity to irritants, and dermatitis develops. A chronic eczema, such as is seen with varicose veins, seems to lower the resistance of the rest of the skin and render the patient more liable to contact dermatitis.

### DIAGNOSIS

The discovery of the irritant responsible for the rash is obviously of paramount importance as treatment is unlikely to be successful if the irritant is still affecting the skin. The history is a very important part of the investigation of the case and may reveal, almost at once in some cases, the cause of the trouble. A rash occurring on areas of skin which have recently been in contact with a known irritant is likely to have been caused by that substance, for example a new washing powder.

An accurate diagnosis can frequently be made after a careful evaluation of the answers to the following questions:

*When did the rash appear?* This may be at a certain time or day each week. For example a patient woke at five o'clock each Sunday morning with intense irritation of the hands and face. Later a rash developed in these areas, and further questioning elicited the fact that she cut off the dead leaves of a number of primulas every Saturday night. She has since avoided primulas and has had no further trouble.

*Where did the rash first occur?* For example a generalized rash may have spread from the areas on the thighs in contact with nickel suspender fastenings and the patient, unless specifically asked about it, may not think the site of origin is important.

*Is there improvement when a certain potential irritant is avoided?* For example dermatitis due to lipstick will usually clear up quickly when the sufferer ceases to use it.

*What sites are affected?* As mentioned above, a rash on the thighs in areas in contact with suspenders is likely to be due to the nickel fastenings. A dermatitis exactly limited in its early stages to the area of the application of a medicament, for example sulphonamide paste, is presumably due to that substance. Dermatitis due to dusts or gases will affect the exposed surfaces and may cease abruptly at the collar or sleeves.

The sites affected by the constitutional eczemas tend to conform to certain patterns. The infantile type occurs chiefly on the cheeks and forehead, the bends of the arms and behind the knees. Seborrhoeic eczema has a predilection for the scalp, eyebrows, ears, front and back of the chest, groins and axillae. Nummular eczema has discrete patches chiefly on the limbs. If the distribution does not conform to one of the patterns of the constitutional eczemas an external irritant should be suspected.

*Is the patch test positive?* The patch test is performed by applying some of the suspected substance to the unaffected skin, commonly the upper arm or back. Tables giving suitable dilutions for testing should be consulted (Sulzberger,

1943). If a liquid is to be used a few drops should be placed on a small piece of lint one inch square and then applied to the skin. A powder can be dusted on to the lint which has previously been moistened. The patch is strapped on with elastoplast and a control patch, omitting the substance to be tested, is placed on another area of skin. At the end of twenty-four hours the patches are removed. Erythema or vesiculation of the area in contact with the suspected chemical is recorded as a positive reaction, and is strong evidence that the substance tested has caused the dermatitis. Occasionally the reaction is delayed and the area should be examined again at the end of forty-eight and seventy-two hours. A negative reaction does not always exclude the tested substance because other factors, such as friction and degreasing agents used to remove the irritant, do not operate in the patch test. This test is not usually done in the acute stage of dermatitis as it may, with some irritants, produce a widespread exacerbation.

### TREATMENT

The first consideration in the treatment is to avoid the offending irritant. This may mean a change of occupation. A housewife whose skin is intolerant to the alkaline soap powders is a great problem as any soap, or even water alone, will aggravate the dermatitis. In some cases rubber gloves, well powdered with talc inside, are helpful; but many people find that the sweating and maceration of the skin, which they cause, preclude their use. Barrier creams such as Rosalex or Innoxa are of little value in preventing further attacks of dermatitis although they help to protect the healthy skin. Desensitization of the skin to irritants is practically impossible. Sedatives such as phenobarbitone, which often work better when combined with aspirin, are helpful. The antihistamine drugs by mouth may help to relieve the irritation.

The essential part of the local treatment is to use bland remedies. In the acute stage normal saline, lead lotion, or one-half per cent silver nitrate compresses are helpful. These must be kept moist by frequent changing. If there is not much exudate Cremor Calamine Co. B.P.C., with one per cent phenol added, is a useful preparation. When the acute stage is passed an application with some covering power is suitable, and a paste such as the following is commonly used: Zinc oxide and starch one drachm of each, yellow soft paraffin two drachms, and hydrous emulsifying ointment B.P. to one ounce. This is applied once or twice a day direct to the skin and covered lightly to protect the clothing. For infection two per cent of ammoniated mercury may be added to the paste and for irritation two to four per cent of crude coal tar. The skin should not be cleansed between applications as the trauma retards healing. Once the acute phase is over superficial X-ray therapy can be given, a suitable course being four doses of *100r* each at intervals of seven to fourteen days.

The prognosis is affected by a number of factors. Once dermatitis has developed continued exposure to the irritant tends to increase the severity of the attack, and also polyvalent sensitivity may supervene. It is therefore, very important that other work shall be found for the workman with occupational contact dermatitis, and assistance obtained for the housewife for many of the household duties. The psychological aspect is of great importance. A period of enforced idleness allows the patient too much time to think of his skin trouble, and to worry about his future capability for work or other duties. This undoubtedly has a bad effect on recovery.

### SUMMARY

Contact dermatitis is common in industry and elsewhere. The essentials in the management of a case are to discover the irritant responsible as soon as possible and to avoid it. Bland local treatments are indicated with superficial X-ray therapy in certain cases. Attention must be paid to the psychological problems which may arise.

### REFERENCES

Sulzberger, M. B. (1943). *Year Book of Dermatology*, Chicago.

